# Design, characterization, and *in vitro* evaluation of magnetic carboxymethylated β-cyclodextrin as a pH-sensitive carrier system for amantadine delivery: a novel approach for targeted drug delivery

**DOI:** 10.1039/d4ra06269h

**Published:** 2025-01-03

**Authors:** Mehrdad Hadadian, Reza Allahyari, Behnam Mahdavi, Esmail Rezaei-Seresht

**Affiliations:** a Department of Chemistry, Faculty of Science, Hakim Sabzevari University Sabzevar Iran b.mahdavi@hsu.ac.ir

## Abstract

In this study, a magnetic carboxymethylated β-cyclodextrin (Mag/CM-β-CD) was developed as a carrier system to assess its capability on drug delivery application by forming an inclusion complex with amantadine (Amn) as a drug model. The synthesized inclusion complex (Mag/CM-β-CD/Amn) was analyzed using various techniques, including FT-IR, XRD, BET, TGA, TEM, VSM, and DLS. The encapsulation efficiency and drug release study of Mag/CM-β-CD/Amn were adopted using the spectroscopic method. Furthermore, the kinetics of drug release by different mathematical models was studied. The cytotoxicity evaluation of Mag/CM-β-CD and Mag/CM-β-CD/Amn was studied using MTT assay against the HUVEC cell line. The TEM imaging showed a spherical morphology and average size of less than 25 nm for the drug complex. Mag/CM-β-CD showed high EE% by absorbing 81.51% of amantadine. Mag/CM-β-CD/Amn showed a pH-sensitive manner with a higher release rate at acidic pH. In addition, a kinetic study reveals that the release process followed the Fickian mechanism and was governed by diffusion. The MTT assay demonstrated low toxicity for the Mag/CM-β-CD/Amn complex in HUVEC cells, showing a cell viability of 57.13% at a concentration of 1000 μg mL^−1^. The results indicate that the developed system is an effective vehicle for transporting drugs in targeted drug delivery applications.

## Abstract 2

In this study, a magnetic carboxymethylated β-cyclodextrin (Mag/CM-β-CD) was developed as a carrier system to assess its capability on drug delivery application by forming an inclusion complex with amantadine (Amn) as a drug model.

## Graphical abstract

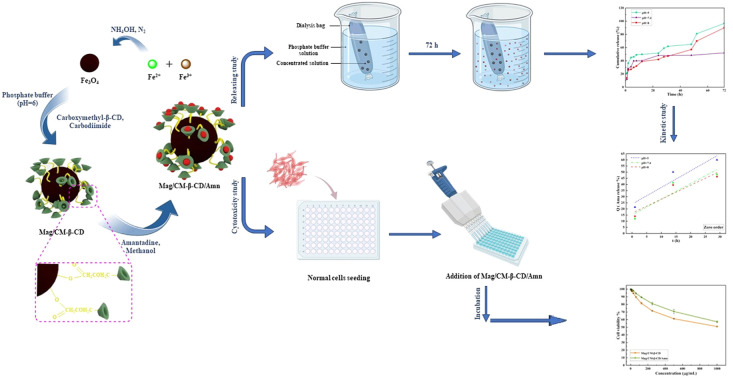

## 1 Introduction

Over the past few years, numerous research studies have focused on enhancing treatment approaches and transitioning from conventional methods to contemporary techniques to minimize harm to healthy cells and specifically target cells during drug delivery processes.^1^ Nanomaterials based on drug delivery systems could improve drug transport and provide opportunities for active-targeting of drug delivery and controlled release.^2^ Nanotechnology can facilitate delivering the correct drug dose to the right place at the appropriate time inside the body to help the drug concentration and effectiveness during treatment.^3^ Magnetic nanoparticles are known as a new class of carriers for drug delivery systems. Paramagnetic iron oxide nanoparticles (MNPs) are unique due to their characteristics, including nano size, magnetic properties, large surface area, simple preparation process, and low toxicity.^4,5^ MNPs possess the capability to bind with different compounds, enabling customization for specific uses such as magnetic separation, tissue regeneration, MRI, hyperthermia treatment, catalysis, molecular diagnostics, and drug delivery systems.^1,6^ have been extensively researched for their ability to deliver drugs to specific organs within the body when subjected to an external magnetic field.^3^ In recent years, polymer-based magnetic nanoparticles, especially cyclodextrins, have gained much attraction for targeting drug delivery.^7^ Recently, polymer-based magnetic nanoparticles, particularly those incorporating cyclodextrins, have garnered significant interest for targeted drug delivery. For example, a folic acid-conjugated glycodendrimer featuring a magnetic β-cyclodextrin core has been developed as a pH-responsive system for the targeted delivery of doxorubicin and curcumin,^7^ Additionally, pH-responsive β-cyclodextrin-assembled Fe_3_O_4_ nanoparticles have been utilized for the selective loading and targeted delivery of stereoisomeric doxorubicin and epirubicin.^8^ Furthermore, Fe_3_O_4_@SiN nanoparticles, where Fe_3_O_4_ serves as the core and SiO_2_ as the shell, have been functionalized with β-cyclodextrin for thymol drug delivery. Another innovative approach involves the fabrication of β-CD-PDA-MNPs through the surface coating of Fe_3_O_4_ nanoparticles with polydopamine, followed by functionalization with 6-thio-β-CD and diclofenac as a model drug.^9,10^ Lastly, CD-GAMNPs were engineered for targeted anticancer drug delivery of retinoic acid by grafting cyclodextrin onto Arabic gum-modified magnetic nanoparticles (GAMNPs) using hexamethylene diisocyanate as a linker. These developments illustrate the versatility and potential of magnetic nanoparticles in enhancing drug delivery systems, particularly in targeting specific tissues while minimizing side effects.^11^

These developments illustrate the versatility and potential of magnetic nanoparticles in enhancing drug delivery systems, particularly in targeting specific tissues while minimizing side effects.

Cyclodextrins (CDs) are natural compounds that are made up of starch by the glycosyl transferase enzyme in bacterial degradation (CGTase).^12^ The most common natural CDs are alpha-, beta-, and gamma-cyclodextrin, which are obtained from six, seven, and eight units of glucose, which are composed of α-(1,4) glycosidic bonds.^13^ CDs have truncated cone shapes with hydrophobic internal cavities and hydrophilic outer surfaces.^14^ CDs can accept lipophilic guests with the correct size in the cavity and form inclusion complexes.^15^ CDs can improve solubility and stability, prevent volatility, mask flavors, and control the release of the guests.^15–18^ CDs can also improve various pharmaceutical qualities, such as drug bioavailability, stability, solubility, and dissolution rate.^19–21^ CDs are prominent tools for innovation in drug delivery systems.^3^ Among cyclodextrins, β-CD is the most popular compound because of its suitable cavity size, reasonable price, non-toxicity, availability, and biodegradability.^22,23^

To date, many studies have been conducted to introduce a magnetic composite with a facile route of preparation and substantial targeting drug delivery benefits. Among many composites, magnetic chitosan, cellulose, and hydrogel have gained more attention over the last decades.^24–26^ However, magnetic cyclodextrin composites with their extraordinary structure may be considered as the better drug carrier due to their ability to form non-covalent interactions with a wide range of hydrophobic drugs, which results in the improved solubility, stability, and transport of lipophilic drugs. However, CDs provide a more controlled release, which is so advantageous when their magnetic composites have been guided to a local site to release a specific dosage of a bioactive substance at a particular time.^27,28^

**Scheme 1:**
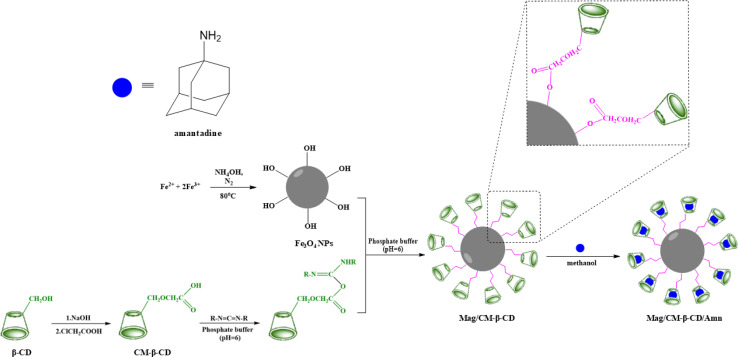
The procedure for preparing the inclusion complex of amantadine with Mag/CM-β-CD.

Amantadine (Scheme 1) is a white, odorless, crystalline powder of the adamantane class of drugs.^29,30^ In October 1966, amantadine was approved by the US Food and Drug Administration (FDA) specifically for the treatment of Asian influenza, and in 1976, it received approval to prevent influenza A.^31^ However, the FDA coincidentally discovered that amantadine can be used to improve Parkinsonian symptoms. Also, it can be used as monotherapy or in combination with levodopa to control symptoms of Parkinson's disease.^30^ It is also known to augment dopamine release and delay its reuptake.^32,33^

The existing literature lacks reports on the formation of an inclusion complex between adamantine and magnetic carboxymethylated β-cyclodextrin. This study introduces a novel magnetic nanocarrier system aimed at enhancing targeted drug delivery. We achieved this by functionalizing the surface of Fe_3_O_4_ nanoparticles with carboxymethyl-β-cyclodextrin (CM-β-CD), creating a nanocarrier that demonstrates “smart” characteristics, allowing for precise control over drug loading and release. Amantadine was selected as a model drug to assess these functionalities. To thoroughly characterize the magnetic nanocarrier's structure and composition, we utilized a comprehensive suite of analytical techniques. Transmission electron microscopy (TEM) provided insights into particle size and morphology, while Brunauer–Emmett–Teller (BET) analysis assessed surface area. Fourier-transform infrared spectroscopy (FT-IR) was employed for functional group identification, and thermogravimetric analysis (TGA) evaluated thermal stability. Magnetic properties were examined using vibrating sample magnetometry (VSM), dynamic light scattering (DLS) analyzed size distribution in solution, and X-ray diffraction (XRD) determined crystal structure. The drug release profile of amantadine from the nanocarrier was meticulously investigated using UV-visible spectrophotometry, followed by the application of mathematical models to elucidate release kinetics and mechanisms. Furthermore, we evaluated the cytotoxicity of the developed nanocarrier against normal human umbilical vein endothelial cells (HUVECs) using the MTT assay, ensuring its biocompatibility. This research not only fills a significant gap in the literature but also contributes to advancing the field of targeted drug delivery systems through innovative magnetic nanocarrier technology.

## 2 Experimental

### 2.1 Materials

FeCl_2_·6H_2_O, FeCl_3_·6H_2_O, HCl, NH_3_, NaOH, and monochloroacetic acid were purchased from Merck (Germany); amantadine (purity >98%), β-cyclodextrin (β-CD), and carbodiimide were sourced from Sigma-Aldrich. The other reagents and chemicals used were of analytical purity.

### 2.2 Instrumentations

XRD patterns were recorded using an X-ray diffractometer (STOE, Germany) with Cu Kα radiation at a voltage of 45 kV and a current of 40 mA, scanning the diffraction angle (2*θ*) from 6° to 90° at a rate of 2° min^−1^. The morphology of the samples was assessed using transmission electron microscopy (EM 208S) at an accelerating voltage of 100 kV. FT-IR spectra were obtained with an ATR PerkinElmer Spectrum IR Version 10.6.2 over the range of 400–4000 cm^−1^. Specific surface areas and pore size distributions were measured using an ASAP 2010 analyzer (Micromeritics, USA). Thermal analysis was conducted with a thermal gravimetric analyzer (TG 209 F3) at a heating rate of 10 °C min^−1^ from 30 °C to 600 °C. The size distribution of nanoparticles was determined using a dynamic light scattering (DLS) instrument (Microtrac, USA). Magnetization measurements were performed with a vibrating sample magnetometer (VSM; Model 7400, Lakeshore Company, USA) at room temperature, while UV-VIS analysis was carried out on a UV-Vis spectrophotometer (Jena Speko L2000) in the range of 200 to 500 nm.

### 2.3 Synthesis of Fe_3_O_4_ NPs

An aqueous solution (50 mL) of ferric chloride (10 mmol) and ferrous chloride (4.26 mmol) was refluxed for 30 min under nitrogen at 85 °C. After 15 min from the zero-time, 7.5 mL of ammonia solution (27%) was poured dropwise for 3 minutes. The color was changed to black immediately. Eventually, an external magnet was applied to collect the precipitates and washed 3 times by deionized water and ethanol. Finally, the precipitates were dried at room temperature.^34^

### 2.4 Preparation of carboxymethylated β-CD (CM-β-CD)

The carboxymethylated β-cyclodextrin was prepared according to a method that was reported previously.^35^ β-CD (2.0 g, 1.76 mmol) and NaOH (1.87 g, 46.5 mmol) were dissolved in 7.4 mL of water at room temperature. Then, 5.4 mL of monochloroacetic acid solution (16.2%) was added to the mixture and stirred at 50 °C for 5 h. After reaching ambient temperature, the pH was adjusted (6–7) by adding HCl. Then, an excess amount of methanol was poured into a neutralized solution at room temperature to precipitate the yield. The white residues were filtered and placed under a hood to dry for 24 hours.

### 2.5 Preparation of Mag/CM-β-CD

The magnetic drug carrier was synthesized according to a reported method.^4^ A 100 mg of Fe_3_O_4_ was suspended in phosphate buffer (2.0 mL, 0.003 M phosphate, 0.1 M NaCl, pH 6) and put in an ultrasonic bath for 10 min at room temperature. Then, carbodiimide solution (0.5 mL, 0.025 g mL^−1^ in phosphate buffer, pH = 6) was added and sonicated for 15 min at room temperature. Next, CM-β-CD solution (2.5 mL, 150 mg in 3 mL phosphate buffer, pH = 6) was added and sonicated for an extra 90 min at RT. Finally, the resulting magnetic nanoparticles were separated by an external magnet, rinsed five times with phosphate buffer, and dried in a vacuum oven for 24 hours.

### 2.6 Loading of amantadine

The Mag/CM-β-CD/Amn complex was synthesized using a co-precipitation.^36^ A 10 mL methanolic solution of amantadine (0.1 g, 0.66 mmol) was stirred for 15 min at RT. After reaching equilibrium, 100 mg of Mag/CM-β-CD was poured and shaken for 24 h at RT. Then, the mixture was sonicated for 30 min and refrigerated for 12 h. The precipitated magnetic complex was separated using an external magnet, and after washing with methanol, it was placed in a vacuum oven at 40 °C for 24 h to dry.

To obtain more yields, all procedures for the synthesis of Mag/CM-β-CD, Mag/CM-β-CD/Amn, and drug release were repeated at least 3 times. The results confirm the reproducibility of all assays.

### 2.7 Determination of loading content of drug in Mag/CM-β-CD/Amn

100 mg of Mag/CM-β-CD was dispersed in an amantadine solution (30 mg in 3 mL methanol), shaken for 24 h, and then placed in an ultrasonic bath for 30 min at RT. Next, the Mag/CM-β-CD/Amn were separated using an external magnet. The encapsulation efficiency (EE%) and loading content (LC%) of Mag/CM-β-CD/Amn were determined by analyzing the unloaded concentration of amantadine using UV-Vis spectroscopy at 286 nm and the following equations:^37^1
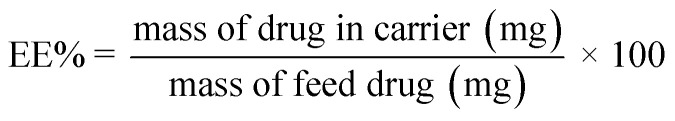
2
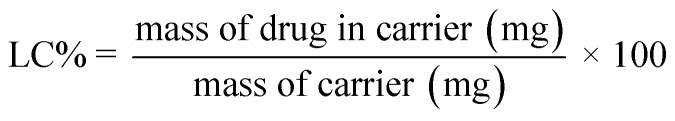


### 2.8 *In vitro* drug release study

The releasing process of amantadine was performed according to a previous study.^38^ For this purpose, a dialysis bag consisting of Mag/CM-β-CD/Amn (50 mg/5 mL PBS) was placed in the containers consisting of 200 mL PBS at different pH (5, 7.4, 8) stirred at 37 °C at 300 rpm. The solution absorbance was read at various times (1, 2, 4, 8, 12, 24, 48, and 72 h). For this purpose, 3 mL of the prepared mixture was withdrawn and replaced by the same volume of fresh PBS after measuring the absorbance at 267 nm. The experiments were performed three times.

### 2.9 Kinetic studies of the drug release

Mathematical studies were adopted to investigate the amantadine release mechanism from Mag/CM-β-CD/Amn. For this purpose, 60% of experimental data obtained from the drug release step was fitted with kinetic models (zero-order eqn (3), first-order eqn (4), Higuchi eqn (5), and Korsmeyer–Peppas eqn (6)), and their curves are plotted.

3 *Q* = *K*_0_*t*

4 Ln *Q* = Ln  *Q*_0_ − *K*_1_*t*

5 *Q* = *K*_H_*t*^1/2^

6 Ln *Q* = *n* Ln *t* + Ln *K*_P_

where *Q* is the percentage of released amantadine at time *t*; *n* is the release exponent; *K*_0_, *K*_1_, *K*_H_, and *K*_P_ are the rate constant of eqn (3), (4), (5), and (6), respectively.

### 2.10 Cytotoxicity evaluation

The cytotoxicity of Mag/CM-β-CD and Mag/CM-β-CD/Amn against HUVECs cell lines (human umbilical vein endothelial cells) was examined using the MTT assay. First, the cell lines were implanted in a 96-well plate and incubated under 5% CO_2_ at 37 °C for 24 hours. After cells reached 80% confluence, Mag/CM-β-CD and Mag/CM-β-CD/Amn (0–1000 μg mL^−1^) were added to the wells and incubated in the dark humidified environment along with DMSO (0.05% v/v) at 37 °C for 24 hours. Later, the cell layers were washed with PBS and incubated with 50 μL of the MTT solution (2 mg mL^−1^) for another 4 hours under the same conditions. Therefore, the cell cultures were centrifuged to remove extra supernatants. Finally, the blue-violet MTT crystals were dissolved by adding 200 μL of DMSO and 25 μL of Sorenson, respectively. The absorbance of each cell culture was measured spectroscopically at 570 nm. The absorbance of the control group was considered 100%. The treatments were assayed three times in independent experiments. The cell viability percentage was obtained from eqn (7):7
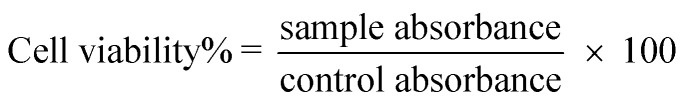


## 3 Results and discussion

The capability of CDs in the formation of inclusion complexes is one of the most important aspects that makes the compounds desirable in the supramolecular chemistry field. CDs with different cavity sizes can form typical complexes, namely host–guest complexes, with various materials, including organic and inorganic compounds.^39^ In this type of complex, the binding of the guest to the host molecules is not a covalent bond.^40^ In addition to the importance of size, the binding strength of the complex depends on the interactions between the surface atoms of host and guest molecules.^41^ The formation of Mag/CM-β-CD/Amn is presented in Scheme 1. According to the scheme, the preparation of the Mag/CM-β-CD/Amn consists of three steps including (1) the formation of CM-β-CD by reaction of β-CD with NaOH and then by monochloroacetic acid; (2) the establishment of the linkage of CM-β-CD to Fe_3_O_4_, in this step, carbodiimide was used to activate the OH group of the carboxymethyl group of cyclodextrin to become a better-leaving group while the OH group of the surface of magnetite particles attacks for the formation of Mag/CM-β-CD; (3) the loading of Amn in the carrier using the co-precipitation method.

### 3.1 Physicochemical characterization of products

#### 3.1.1 FT-IR analysis

**Fig. 1:**
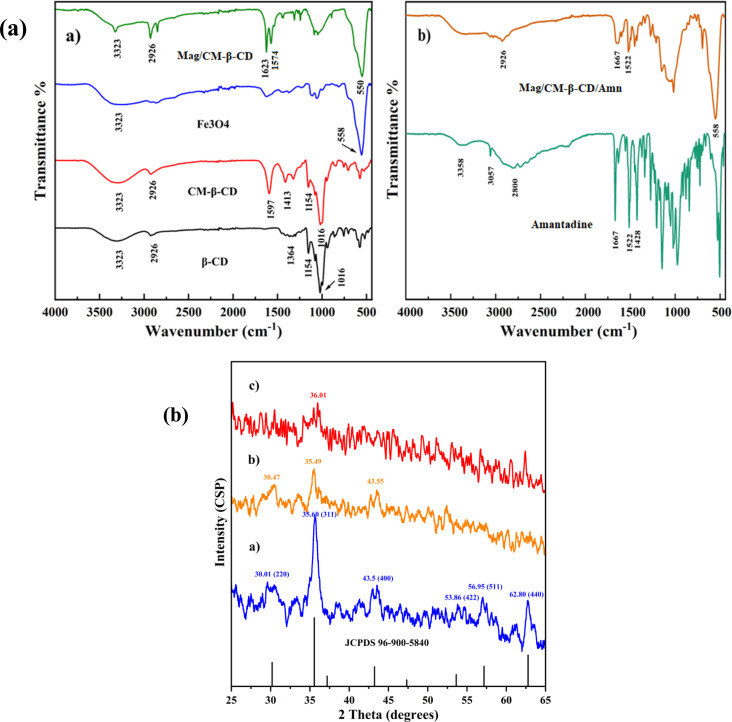
(a) FT-IR analysis of (a) β-CD, CM-β-CD, Fe_3_O_4_, Mag/CM-β-CD, and (b) Amn, and Mag/CM-β-CD/Amn and (b) the XRD patterns of (a) Fe_3_O_4_, (b) Mag/CM-β-CD, and (c) Mag/CM-β-CD/Amn.

Fig. 1a presents the FT-IR spectra of β-CD, CM-β-CD, Fe_3_O_4,_ and Mag/CM-β-CD. For β-CD, two bands at 1016 and 1154 cm^−1^ were assigned to stretching vibrational bands of C–O. The prominent band at 3323 cm^−1^ was related to the hydroxyl bond (O–H). Furthermore, the band at 2926 cm^−1^ belonged to the aliphatic C–H bond. For the prepared CM-β-CD, a new peak at 1597 cm^−1^ related to the C=O bond of the carboxymethyl group, along with all peaks of pristine β-CD, approved the successful formation of CM-β-CD. The band shift to a low wavenumber could be attributed to the carboxylate anion form of the carboxyl functional group due to the use of sodium hydroxide to prepare CM-β-CD.^42^ The spectrum of Fe_3_O_4_ showed a band at 558 cm^−1^ assigned for Fe–O bond, the other one at 3400 cm^−1^ belonged to the O–H bond, which was found at the surface of the synthesized Fe_3_O_4_ and indicated the successful synthesis of Fe_3_O_4_.^26^ All the mentioned bands for CM-β-CD and Fe_3_O_4_ were found in the Mag/CM-β-CD spectrum with little shifts. Besides, the peaks at 1623 cm^−1^ and 1574 were related to the bounded and unbounded C=O of the carboxyl group. The presence of both CM-β-CD and Fe_3_O_4_ characteristic peaks in Mag/CM-β-CD and a small shift in the C=O bond from 1597 cm^−1^ to 1623 cm^−1^ confirmed the successful synthesis of Mag/CM-β-CD.

The spectra of amantadine and Mag/CM-β-CD/Amn are exhibited in Fig. 1b. The spectrum of amantadine showed two peaks at 3358 and 1522 cm^−1^ for the stretching and bending vibrations of the N–H bond, respectively. The peak at 1667 cm^−1^, belonged to the stretching vibrational band of C–N. Also, the bending vibrational band of CH_2_ groups is found at 1428 cm^−1^. The band at 2800 cm^−1^ was related to the stretching vibrational band of CH. The obtained FT-IR spectrum of Amn was similar to a previous report.^43^ The FT-IR spectrum of Mag/CM-β-CD/Amn showed different peaks at 2926 (CH), 1667 (CN), 1522 (NH), and 558 (FeO) cm^−1^, and the broad peak at 3300–3500 cm^−1^ belonged to OH and NH bonds. The presence of both amantadine and Mag/CM-β-CD characteristic peaks in the FT-IR spectrum of Mag/CM-β-CD/Amn with small changes in shape and intensity approved successful loading of amantadine in the prepared Mag/CM-β-CD carrier.

#### 3.1.2 XRD analysis

The crystallinity of Fe_3_O_4,_ Mag/CM-β-CD, and Mag/CM-β-CD/Amn was investigated using the X-ray diffraction technique. The results are exhibited in Fig. 1. The XRD pattern of pure Fe_3_O_4_ (Fig. 1a) shows six characteristic signals at 2*θ* values of 30.12, 35.60, 43.52, 53.86, 56.95, and 62.80, which belong to the planes of (220), (311), (400), (422), (511), and (440), respectively. These data show that the synthesized Fe_3_O_4_ has a cubic structure according to the JCPDS card (ref. no. 96-900-5840) standard and is in good agreement with magnetic particles (Fe_3_O_4_) that were reported previously.^44,45^ As can be seen in the diffractogram, the synthesized Fe_3_O_4_ has a crystalline nature. The X-ray pattern of Mag/CM-β-CD (Fig. 1b) depicts three diffraction signals at the values of 30.47°, 35.49°, and 43.55°, which are similar to those of pure Fe_3_O_4_ with some modification. This observation confirms the linkage of Fe_3_O_4_ to CM-β-CD; however, the peak intensity has been reduced. From the Mag/CM-β-CD/Amn XRD pattern (Fig. 1c), the main characteristic peak of Fe_3_O_4_ is still visible in the resultant magnetic nanoparticles, with shifting to 36.01° at an angle of 2*θ*. Besides, the crystalline nature of yield is completely different compared to the starting materials. The XRD pattern of Fe_3_O_4_ was completely changed after modification with CM-β-CD and loading of amantadine. These changes in peaks' presence, shape, and intensity exhibit the successful preparation of Mag/CM-β-CD/Amn in a new solid crystalline phase.^46^

#### 3.1.3 Nitrogen adsorption/desorption isotherm (BET analysis)

**Fig. 2:**
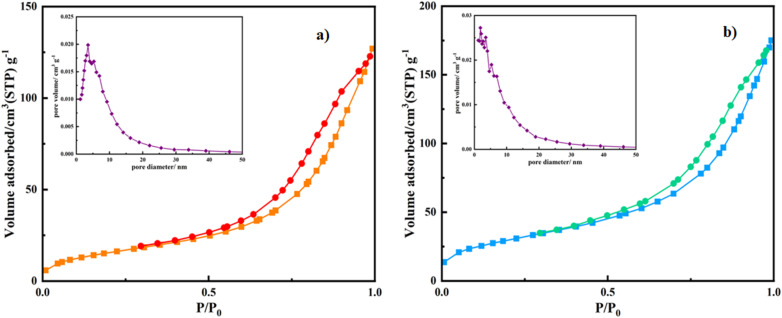
Nitrogen adsorption–desorption isotherms and Barrett–Joyner–Halenda (BJH) plots of (a) Mag/CM-β-CD and (b) Mag/CM-β-CD/Amn.

Brunauer–Emmett–Teller (BET) surface (*S*_BET_) area analysis is a sufficient technique to study the size and pores of carriers for drug delivery systems. These characters show a substantial impact on the drug encapsulation process.^47,48^ In the present study, BET analysis was run along with the FT-IR technique to acquire more evidence about the successful loading of amantadine molecules in the Mag/CM-β-CD mesh. The porous features of Mag/CM-β-CD/Amn and Mag/CM-β-CD are represented in Fig. 2. The *S*_BET_ values and pore volume of Mag/CM-β-CD (59 m^2^ g^−1^, 0.19 cm^3^ g^−1^) and Mag/CM-β-CD/Amn (108 m^2^ g^−1^, and 0.27 cm^3^ g^−1^) were measured. An increase in values was observed after loading amantadine molecules in Mag/CM-β-CD. This phenomenon may be correlated to the formation of new pores after inserting amantadine in the magnetic carriers, resulting in more steric hindrance.^49^ The mean pore diameter of Mag/CM-β-CD/Amn is relatively smaller than the pore diameter of pure Mag/CM-β-CD by 9.896 and 13.24 nm, respectively. This observation indicates that the entrapment of drug molecules in the Mag/CM-β-CD pores happened successfully. Besides, N_2_ adsorption/desorption plots of Mag/CM-β-CD and Mag/CM-β-CD/Amn show IV isotherm, indicating the mesoporous structure.^7^ It seems the formation of Mag/CM-β-CD was applied as a complex magnetic network. So, the entrance of Amn molecules into the network causes the increase of the gap between the network units and leads to an increase in the *S*_BET_ and total pore volume of the product.

The BET analysis of Mag/CM-β-CD has been compared to the other magnetic carriers, which were reported previously (see Table 1). Among the carriers, a magnetic metal–organic framework conjugated with β-CD (Fe_3_O_4_-SiO_2_-MIL-100 (Fe)-β-CD) as a drug delivery system.^3^ A polymer of β-CDs (β-CD-P) was synthesized using pyromellitic dianhydride to link the surface of Fe_3_O_4_ nanoparticles (Fe_3_O_4_@β-CD-P NPs). Additionally, Fe_3_O_4_-β-CD and Fe_3_O_4_-β-CD-CTD-FA were developed as magnetic nanocarriers, where citric acid and triazine dendrimer were employed to attach folic acid to the surface of Fe_3_O_4_-β-CD. In comparison, Mag/CM-β-CD demonstrated a significantly higher BET surface area and pore volume than the other carriers. The increased porosity enhances drug encapsulation capabilities, which is crucial for improving the efficiency of drug delivery systems. Furthermore, this property indicates that Mag/CM-β-CD could serve as an effective adsorbent in other applications, such as water and air purification.

**Table 1:** The BET parameters comparison between the prepared Mag/CM-β-CD and different magnetic carriers was reported in the literature

Carrier	BET surface area (m^2^ g^−1^)	Total pore volume (cm^3^ g^−1^)	Average pore size (nm)	References
Mag/CM-β-CD	59	0.19	13.42	Present research
Fe_3_O_4_@β-CD-P NPs	9.5	0.038	3.3	50
Fe_3_O_4_-β-CD	4.34	0.00807	7.4	7
Fe_3_O_4_-β-CD-CTD-FA	29.585	0.049	6.6	7
Fe_3_O_4_-SiO_2_-MIL-100 (Fe)-β-CD	6.071	0.03509	23.12	3

#### 3.1.4 Determination of magnetic properties

**Fig. 3:**
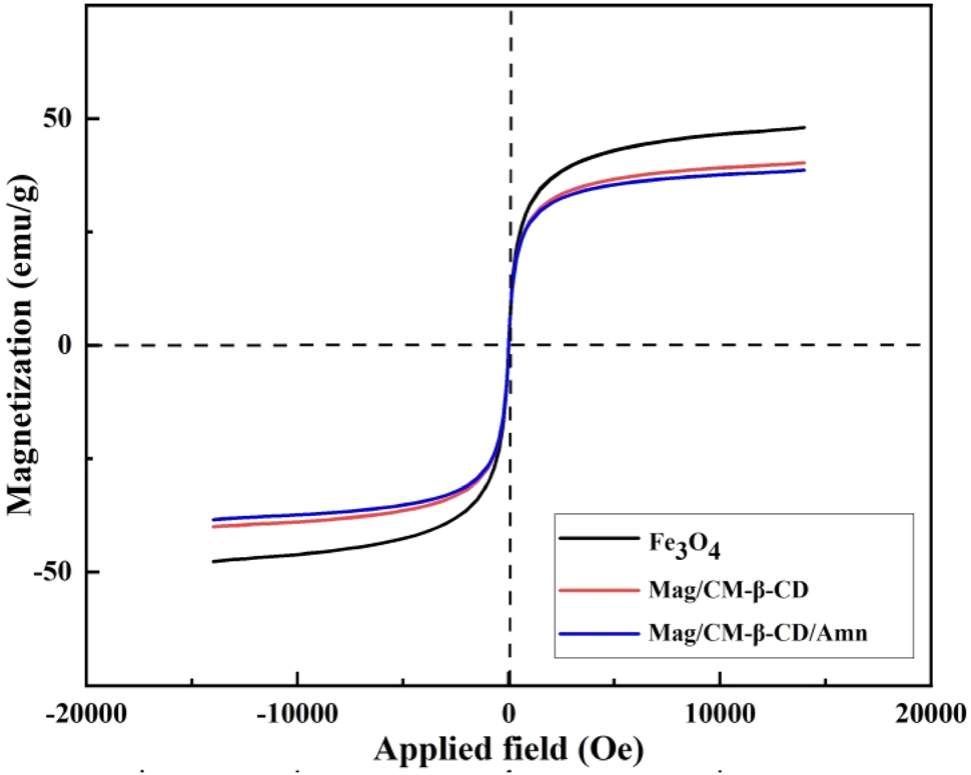
VSM magnetization curve of Fe_3_O_4_, Mag/CM-β-CD, and Mag/CM-β-CD/Amn.

The magnetization properties of Fe_3_O_4_, Mag/CM-β-CD, and Mag/CM-β-CD/Amn were analyzed using a vibration sample magnetometer (VSM). The results are shown in Fig. 3. The amounts of 48, 40, and 38 were found for saturation magnetization (*M*_S_) values of Fe_3_O_4_, Mag/CM-β-CD, and Mag/CM-β-CD/Amn, respectively. The decrease in (*M*_S_) can be related to the stepwise introduction of dielectric particles to the Fe_3_O_4_ surface. Also, the absence of a hysteresis loop (*B*_r_) and zero coercivity (*H*_c_) signify that the prepared Mag/CM-β-CD/Amn has a superparamagnetic nature, which is caused by nano-size particles.^3^ By coating Fe_3_O_4_ with CM-β-CD shells, the value of *B*_r_ (*y*-intercept where *x* = 0) was decreased. This phenomenon results from reducing the interaction between Fe_3_O_4_ nanoparticles. This is a beneficial feature for Mag/CM-β-CD in biomedical applications by dispersing it in water for hours before precipitation.^3^ So far, a few researches have been carried out on the formation and usage of magnetic carriers in drug delivery systems. A comparison between the obtained results of the present study and the reported ones is given in Table 2, The *M*_S_ value of Mag/CM-β-CD showed better magnetic properties in comparison with magnetic cyclodextrin/maltodextrin nanosponge (magnetic NSs), mefenamic acid-loaded magnetized seed hydrogel (Fe/POSH/MFA),^26^ amine-functionalized Fe_3_O_4_/microporous silica nanoparticles (Fe_3_O_4_/MSN-NH_2_),^52^ poly-*N*-5-acrylamidoisophthalicacid grafted onto Fe_3_O_4_ magnetic nanoparticles (MNPs@PAIP-CD), and β-CD surface grafted to Fe_3_O_4_@silica@MOF nanocomposite (Fe_3_O_4_@SiO_2_@MIL-100 (Fe)/β-CD). Therefore, the prepared Mag/CM-β-CD can be considered an appropriate drug delivery agent as it can be separated easily by an external magnet.^53^

**Table 2:** The saturation magnetization (*M*_S_) values comparison between the prepared MNPs and different magnetic carriers reported in the literature

Carrier	Saturation magnetization values (emu g^−1^)	References
Mag/CM-β-CD	40	This work
Magnetic NSs	19	37
MNPs@PAIP-CD	19.5	51
Fe_3_O_4_@SiO_2_@MIL-100 (Fe)/β-CD	5.2	3

#### 3.1.5 TEM imaging analysis

**Fig. 4:**
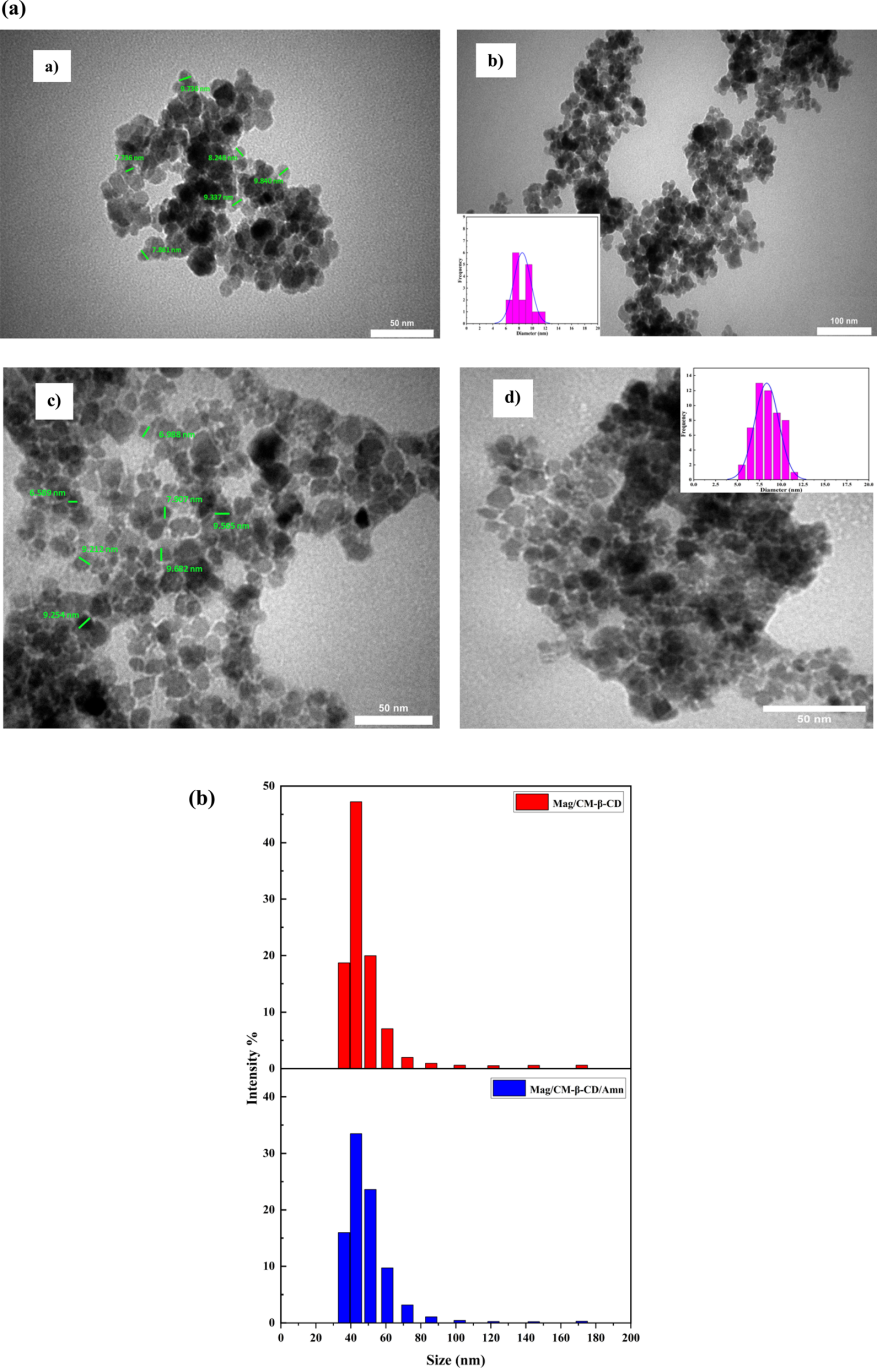
(a) TEM images and PSD of Mag/CM-β-CD (a and b) and Mag/CM-β-CD/Amn (c and d) and (b) particle size distribution of Mag/CM-β-CD and Mag/CM-β-CD/Amn.

Fig. 4a exhibits the TEM images and particle size distribution (PSD) of Mag/CM-β-CD and Mag/CM-β-CD/Amn. The results reveal a spherical shape for the magnetic nanoparticles with a mean diameter of 8.34 and 8.49 nm for Mag/CM-β-CD and Mag/CM-β-CD/Amn, respectively. Moreover, they exhibit a narrow size distribution ranging from 6.8 to 11.1 nm for the carrier and 5.6 to 11.3 nm for the final product. The CM-β-CD layer cannot be seen in TEM images.^4^ Also, it is proved that the magnetic nanoparticles with zero coercivity and remanence that have a size smaller than 30 nm show superparamagnetic properties.^35^ Therefore, the prepared Mag/CM-β-CD/Amn shows this remarkable feature, which is also approved by VSM results. Nanoparticles with spherical shapes are the best candidates for participation in drug delivery systems, as they provide a larger surface area.^7^ The morphology and size of the final product indicate that Mag/CM-β-CD have significant capabilities for utilization as drug carriers.

#### 3.1.6 DLS analysis

To evaluate the hydrophobic diameter of Mag/CM-β-CD and Mag/CM-β-CD/Amn, dynamic light scattering (DLS) analysis was run (Fig. 4b). According to previous reports, a high aggregation of products is an undesirable property in drug delivery systems. However, Mag/CM-β-CD shows appropriate dispersion with an average particle size of 40.40 nm and a polydispersity index (PDI) of 0.0931. Moreover, Mag/CM-β-CD/Amn shows approximately similar particle size distribution with an average of 41.70 nm. Because of the aggregation of Fe_3_O_4_ in water, the diameter of the Mag/CM-β-CD and Mag/CM-β-CD/Amn is quite different from that proven by TEM analysis.^7^

#### 3.1.7 Thermal analysis

**Fig. 5:**
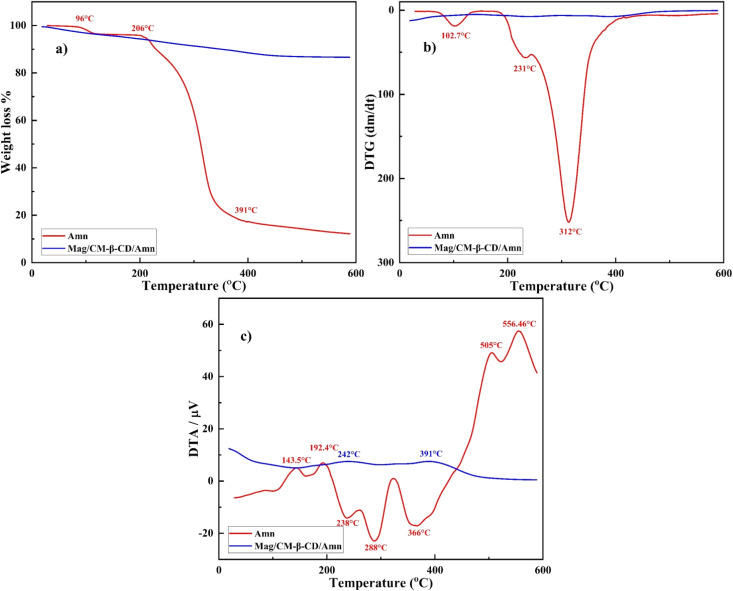
(a) TG%, (b) DTG, and (c) DTA curves of Amn and Mag/CM-β-CD/Amn.

Thermal analysis is an efficient method to investigate thermal stability and further evidence of the formation of the inclusion complex.^54,55^ The thermal analysis of amantadine and the Mag/CM-β-CD/Amn are shown in Fig. 5a–c. As shown in Fig. 5a, an initial weight loss occurred at 96 °C due to moisture loss for amantadine. Then, the decomposition is observed at 206–391 °C, where 77.6% of weight is lost. In contrast, the weight of Mag/CM-β-CD/Amn stood quite constant by increasing in temperature, which reveals the high thermal stability of the prepared complex. Meanwhile, the TGA thermogram of Mag/CM-β-CD/Amn reveals the first weight loss (4.41%) at 100–150 °C due to the loss of physically adsorbed water.^28^ The second weight loss (8.57%), which occurs at 150–420 °C, is related to the decomposition of organic residue on the nanoparticles. Mag/CM-β-CD/Amn indicated good stability in the range of 420–600 °C.

DTG diagram is represented in Fig. 5b. Amantadine shows two minor reactions against temperatures at 102.7 and 231 °C and a maximum reaction at 312.42 °C. DTG profile of Mag/CM-β-CD/Amn displays no detectable decomposition. Also, the DTA graph is exhibited in Fig. 5c, where endothermic peaks of amantadine are placed at 143.5, 192.4, 505, and 556.46 °C, and exothermic peaks are located at 238, 288, and 366 °C. However, Mag/CM-β-CD/Amn shows two minor endothermic peaks at 242 and 391 °C, utterly different from the observed peaks for amantadine. DTG and DTA profiles reveal that the prepared inclusion complex is formed successfully, and there is no evidence of uncomplexed drug molecules in the final product.^56^

### 3.2 Drug loading study

To identify the capacity of Mag/CM-β-CD as a magnetic drug carrier in delivery systems, the co-loading behavior of amantadine on the surface of Mag/CM-β-CD was investigated.^7^ For this case, a pre-determined amount of amantadine was loaded on the Mag/CM-β-CD. The concentration of the remaining drug was obtained by spectroscopic method at 286 nm and compared with the calibration curve of amantadine (10–100 mM). The loading percentage and EE% were found to be 26.9% and 81.51%, respectively. The Mag/CM-β-CD carrier shows a remarkable loading capacity. As the interaction of the host and guest molecules is a non-covalent such as van der Waals forces, hydrophobic interaction, hydrogen binding, and London dispersion forces,^41,57^ different factors affect the high drug loading: (I) strong hydrogen bonding between the amino (–NH_2_) group of amantadine and hydroxyl (–OH) or carboxyl (–COOH) groups of Mag/CM-β-CD. In addition, the p*K*_a_ of amantadine is 10.48, so it has a positive charge in the PBS media (pH = 7.4).^58^ During encapsulation, the –NH_2_ group of amantadine protonated with a positive charge. On the other hand, the –OH and –COOH groups of Mag/CM-β-CD become deprotonated with many negative charges on the surface, which leads to the formation of an electrostatic interaction between amantadine and Mag/CM-β-CD.^7^ (II) The high surface area and total pore volume of Mag/CM-β-CD cause the trapping of amantadine molecules in the pores and spaces of the carrier. (III) The geometrical dimension of the host molecule affects the efficacy of the co-loading process.^59^ As can be seen in Scheme 1, the specific three-dimensional structure of amantadine fit very well with the cavity of CM-β-CD. Accordingly, Mag/CM-β-CD shows high drug encapsulation efficiency.

### 3.3 *In vitro* amantadine release study

**Fig. 6:**
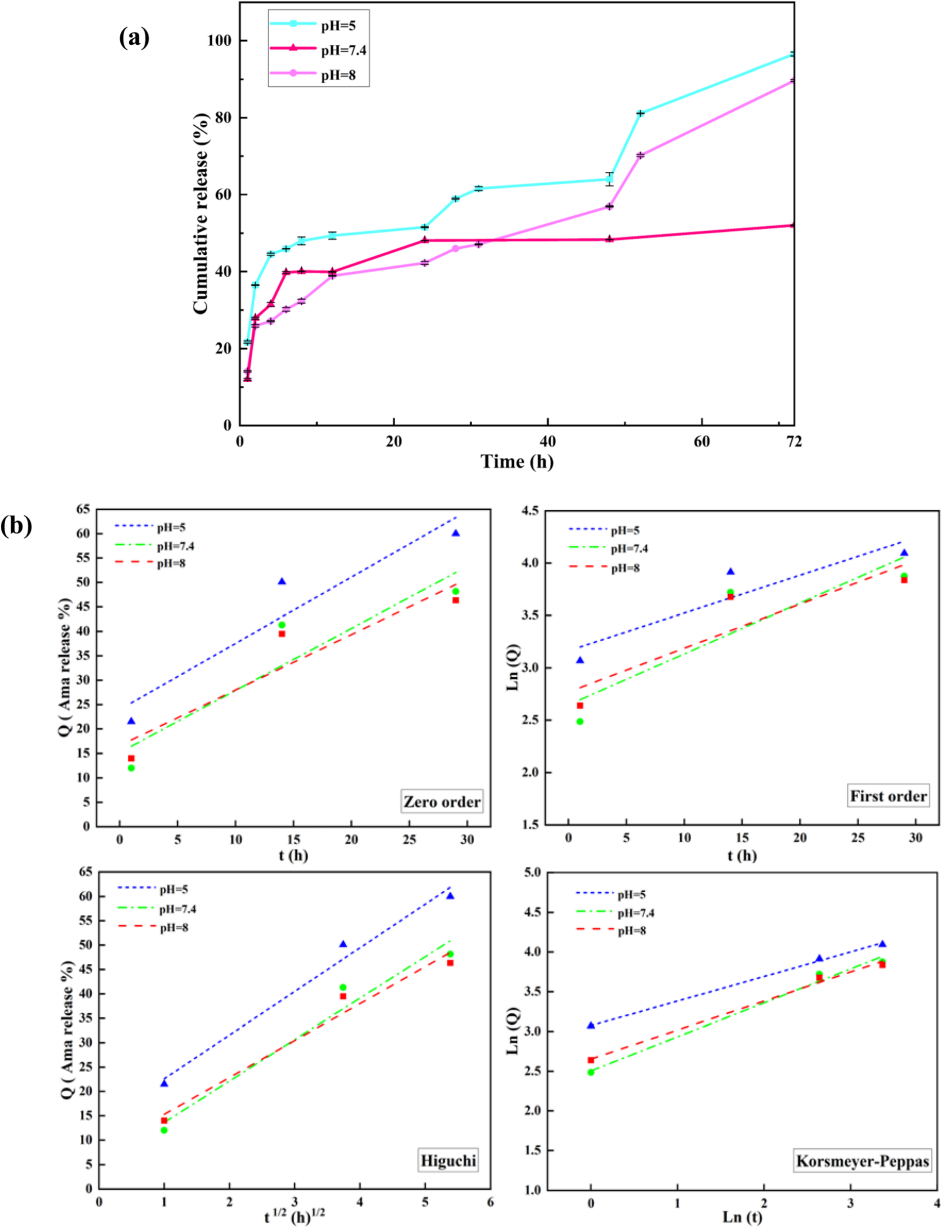
(a) *In vitro* amantadine release profiles from Mag/CM-β-CD in aqueous solution at pH 5, 7.4, and 8 at 37 °C and (b) kinetic models of the drug releases from Mag/CM-β-CD/Amn.

The *in vitro* amantadine release from Mag/CM-β-CD was conducted in different pH values simulated for tumor region pH (5), physiological pH (7.4), and pancreas pH (8) at 37 °C to find the cumulative release of the drug in different parts of the human body and examine the pH responsiveness of prepared magnetic nanocarrier.^60^Fig. 6a illustrates the obtained results for the drug release. The profiles show that nearly 20% of amantadine was released in the first hour in all pHs. This could be related to the desorption of amantadine molecules that are entrapped on the surface of the carrier.^61^ While 96.6%, 90%, and 52% of the drug are released from Mag/CM-β-CD after 72 hours at pH = 5, followed by pH = 8 and pH = 7.4, which are related to amantadine molecules bound to cyclodextrins.^62^ The best result was found for the acidic media because the acidic pH could weaken the π–π interaction between the drug and carrier.^7^ The second result for drug release was obtained for pH = 8 with a value of 90%. This phenomenon may be explained by the specific physical property of β-cyclodextrins containing hydrophobic inner cavities. As the pH moves to 8, the concentration of hydroxide ions has risen in the solution. Consequently, –OH groups were replaced with amantadine molecules in the inner cavity of cyclodextrin due to its higher hydrophobic properties and preferred interaction between two hydrophobic molecules.^40^ Besides, basic hydrolysis can affect the release of drug molecules from CDs in the basic pHs. On the other hand, the degree of protonation depends on the pH value. As the pH moves to acidic or basic values, the degree of protonation increases, which could weaken the hydrogen bondings between the drug and carrier. In this way, the release profile of amantadine is boosted in non-neutral pHs. At acidic pH, the –NH_2_ group of amantadine converts to (NH_3_^+^), so it cannot form hydrogen bonding. Besides, the –COOH and –OH groups of carriers deprotonate to (COO^−^) and (O^−^) cannot participate in the formation of hydrogen bonds.^63^ From this point, the charge of both drug and carrier molecules stood neutral at pH = 7.4. It is assumed that the sustainable release of amantadine from Mag/CM-β-CD (52% after 72 hours) at this pH could result from this phenomenon. Because of the acidic environment of tumors, lysosomes, and endosomes, Mag/CM-β-CD, a pH-responsive drug carrier, is a promising candidate to transport cancer drugs to specific regions and release them with high efficiency.^62^

### 3.4 *In vitro* amantadine release kinetics

To evaluate the kinetic amantadine release, the results of the last section are fitted to standard mathematical equations, which were introduced previously.^64^Table 3 and Fig. 6b represent the calculated kinetic parameters and curves. The comparison of obtained mathematical data of all curves indicates that the Korsmeyer–Peppas model shows proper fitting to experimental results by higher *R*^2^ values in all pHs. In addition, experimental data for release up to 60% was fitted to the Korsmeyer–Peppas equation. The Ln *Q* is plotted as a function of Ln *t* to determine the *n* (exponent of release) values. The *n* values signify the mechanism of drug release, where *n* ≤ 0.43 the release follows the Fickian model; for 0.43 < *n* < 0.85 follows non-Fickian and *n* > 0.85 indicates case II models.^65^

**Table 3:** The kinetic parameters for amantadine release from Mag/CM-β-CD/Amn in different pH values

pH	Zero-order (*R*^2^)	First-order (*R*^2^)	Higuchi (*R*^2^)	Korsmeyer–Peppas	
				*R* ^2^	*n*
5	0.904	0.849	0.983	0.998	0.309
7.4	0.859	0.8	0.961	0.989	0.428
8	0.873	0.817	0.969	0.993	0.365

As can be seen in Table 3, all the obtained *n* values are less than 0.43 at all examined pHs, which corresponds to the Fickian mechanism. The Fickian mechanism is a fundamental concept in drug release that describes the transport of drug molecules through a polymeric medium based on concentration gradients.^66^ The Fickian model describes drug release from carrier systems based on diffusion. In other words, diffusion is the primary mechanism governing drug release in this model. In Fickian diffusion, the rate of drug release is independent of the drug concentration within the carrier. This means that even as the drug concentration decreases in the polymer, the release rate remains constant.^67^

### 3.5 Cytotoxicity assay

**Fig. 7:**
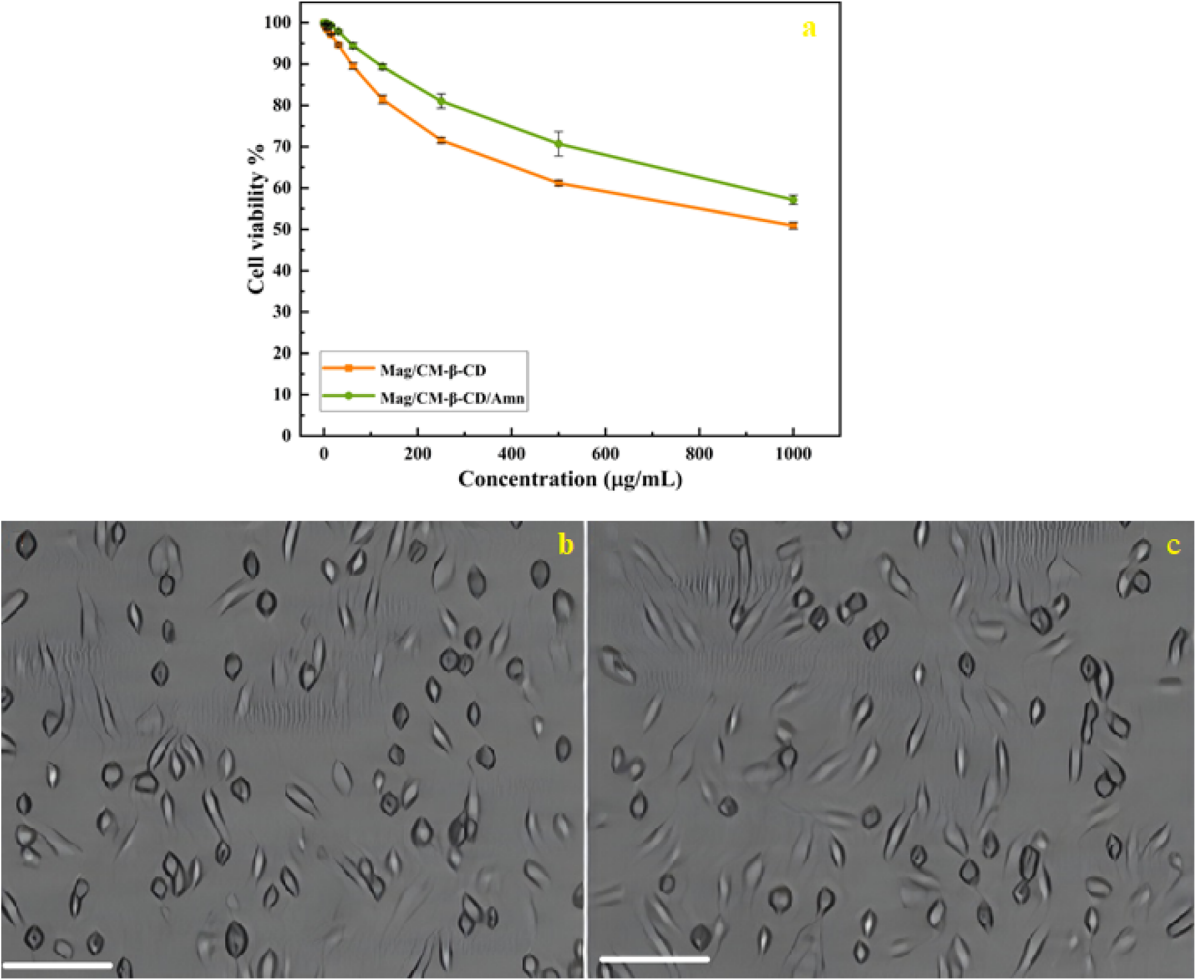
(a) Percent viability measured for HUVECs after treatment with Mag/CM-β-CD and Mag/CM-β-CD/Amn; the images of HUVEC cells treated by the Mag/CM-β-CD (b) and Mag/CM-β-CD/Amn (c) in the MTT assay. Scale bar: 100×.

To evaluate the biocompatibility of the prepared drug carrier and its inclusion complex, the cytotoxicity of Mag/CM-β-CD and Mag/CM-β-CD/Amn were assessed against the normal cells of HUVEC at different concentrations (0–1000 μg mL^−1^). HUVECs are utilized to investigate the toxicity of various metallic nanoparticles.^68^ As shown in Fig. 7a, Mag/CM-β-CD shows no obvious toxicity, even at high concentrations. This observation can be related to the presence of β-CD in the structure of the prepared drug carrier, which is a non-toxic ingredient.^69^ Therefore, Mag/CM-β-CD can be considered a safe and biocompatible carrier in drug delivery systems. Besides, after loading the Amn drug on the Mag/CM-β-CD, the cell viability of the inclusion complex increases to 57.13%. The images of cells treated by Mag/CM-β-CD and Mag/CM-β-CD/Amn are shown in Fig. 7b and c. This observation may be related to the fact that Fe_3_O_4_ has unoccupied orbitals that cause damage to the normal cells. After loading amantadine, these orbitals become occupied by amantadine's free electrons, and the cytotoxicity of the product decreases. The same result was reported in a previous paper.^70^ Moreover, the prepared Mag/CM-β-CD/Amn shows higher cell viability compared to the other drug-loaded magnetic carriers, such as the magnetic composite hydrogel that was fabricated by the graft copolymerization of itaconic acid (IA) onto starch and alginic acid in the presence of graphene sheets and Fe_3_O_4_ nanoparticles for guaifenesin delivery (85% cell viability at 3 μg mL^−1^),^71^ and doxorubicin-loaded core–shell mesoporous silica folic acid-grafted nanocomposite for intracellular enzyme-triggered drug delivery (72% cell viability at 125 μg mL^−1^).^72^ Magnetic Fe_3_O_4_ coated by layered double hydroxide as a methotrexate delivery system for targeted cancer therapy (≈80% cell viability at 120 μg mL^−1^).^73^ The low toxicity of Mag/CM-β-CD/Amn, even at the concentration of 1000 μg mL^−1^, indicates that the prepared inclusion complex is a safe drug to replace instead of amantadine.

### 3.6 Comparison of the present study with previous studies on amantadine

To gain more insights into the advantages of the prepared drug delivery systems for amantadine, which were explored comprehensively in this study, we have compared some of the characteristics of the prepared Mag/CM-β-CD with others. In this way, poly(dl-lactic acid)–methacrylic acid nanospheres bound to the chelating ligand diethylenetriaminepentaacetic acid (DTPA) that was synthesized for targeted delivery of amantadine show substantially lower drug encapsulation by 35.84% compared to Mag/CM-β-CD by 81.51%. The low amount of drug encapsulation efficiency leads to the utilization of a higher amount of prepared drug delivery systems to obtain the recommended dosage of amantadine for appreciable affection. Moreover, the particle size of drug carriers is so critical in these types of materials, and these nanospheres were synthesized with a particle size of 81.94 nm, which is dramatically bigger than that of Mag/CM-β-CD. In addition, the cumulative release of amantadine from these nanospheres records just below 10% after 72 h. Even though this carrier shows a more prolonged drug release compared to the Mag/CM-β-CD, it should be considered that this carrier system can't provide a sufficient dosage of the amantadine in a certain region of the body even after 72 hours.^74^ Moreover, the amantadine-based ion-pair amphiphiles were synthesized using oleic acid surfactant through a proton transfer reaction between amantadine and oleic acid molecules. These vesicles exhibited a broad size distribution, aggregating at 200–300 nm in aqueous solution, which is significantly larger than the 43 nm size of Mag/CM-β-CD/Amn. This aggregation is an undesirable property in drug delivery systems. Furthermore, the drug loading in the vesicles was found to be 34.87%, which is lower than the encapsulation efficiency (81.51%) achieved by Mag/CM-β-CD. Finally, the release rate of ion-pair amphiphile vesicles was much faster compared to Mag/CM-β-CD, with approximately 65% of amantadine being released in PBS within just 2 hours. After this initial burst, there was no significant further release, and the maximum drug release was around 70% after 6 hours. That has no remarkable difference from the free drug.^75^

## 4 Conclusion

In this study, we successfully synthesized magnetic nanoparticles (Fe_3_O_4_) conjugated with CM-β-CD for targeted drug delivery applications, specifically focusing on the encapsulation and release of amantadine. The findings demonstrate that the MNPs exhibit a high encapsulation efficiency of 81.51%, attributed to the high surface area of the carrier and the strong interactions between amantadine and the functional groups on the CM-β-CD surface. The pH-sensitive release profile observed *in vitro* indicates that the MNPs can effectively release the drug in acidic environments, which is particularly relevant for targeting tumor tissues where the pH is often lower than in healthy tissues. The characterization techniques employed, including FT-IR, XRD, TGA, BET, and DLS, confirm the successful formation of the inclusion complex and highlight the favorable physicochemical properties of the synthesized MNPs. The thermal stability of amantadine increased dramatically and showed high particle magnetization. Furthermore, Mag/CM-β-CD/Amn has spherical geometry with a narrow particle size distribution in the nano-sized territory. The superparamagnetic nature of the nanoparticles allows for the potential application of an external magnetic field to enhance targeted delivery, minimizing systemic side effects and improving therapeutic outcomes. Looking forward, the approach of synthesizing magnetic CM-β-CD offers significant opportunities for further development in the field of drug delivery systems. Additionally, the biocompatibility and long-term stability of these carriers in biological systems warrant further investigation to assess their practical implementation in clinical settings. The potential for these designed carriers in medicine is promising, particularly in the treatment of diseases requiring targeted therapy, such as cancer and neurodegenerative disorders. By leveraging the unique properties of magnetic nanoparticles and cyclodextrins, we envision a new generation of drug delivery systems that can provide precise and controlled release of therapeutics, ultimately improving patient outcomes and advancing the field of personalized medicine.

## Data availability

The data presented in this study are available on request from the corresponding author.

## Conflicts of interest

The authors declare that they have no known competing financial interests or personal relationships that could have appeared to influence the work reported in this paper.
